# Effect of sea-bottom elasticity on the propagation of acoustic–gravity waves from impacting objects

**DOI:** 10.1038/s41598-018-37626-z

**Published:** 2019-01-29

**Authors:** Usama Kadri

**Affiliations:** 1 School of Mathematics, Cardiff University, Cardiff, CF24 4AG UK; 2 Department of Mathematics, Massachusetts Institute of Technology, 77 Mass Ave, Cambridge, 02139 MA USA; 3 School of Mechanical and Materials Engineering, University College Dublin, Belfield, Dublin 4, Ireland

## Abstract

Recent analysis of data, recorded on March 8th 2014 at the Comprehensive Nuclear-Test-Ban Treaty Organisation’s hydroacoustic stations off Cape Leeuwin Western Australia, and at Diego Garcia, has led to the development of an inverse model for locating impacting objects on the sea surface. The model employs the phase velocity of acoustic–gravity waves that radiate during the impact, and only considers their propagation in the water layer. Here, we address a significant characteristic of acoustic–gravity waves: the ability to penetrate through the sea-bottom, which modifies the propagation speed and thus the arrival time of signals at the hydrophone station. Therefore, we revisit some signals that are associated with the missing Malaysian Aeroplane MH370, and illustrate the role of sea-bottom elasticity on determining impact locations.

## Introduction

Motivated initially by locating the missing Malaysian Aeroplane MH370, ref.^1^ studied the radiation of acoustic–gravity waves from impacting objects. ref.^1^ presented a technique for locating objects impacting at the sea surface using an inverse approach. Data recorded at hydrophone stations were employed to calculate the location of events not only at the sea surface but also at the sea-bottom (i.e., earthquakes), which is found to be possible since the far-field solution of both problems is very similar. The proposed technique was validated by ref.^1^ by comparing the calculated locations of two earthquake epicentres with existing data from seismometers. Although the agreement was sufficient at such large distances (order of thousands kilometres), discrepancies were still found. These might be associated with the sea-bottom rigidity assumption postulated in the original model. When rigidity is assumed, acoustic–gravity waves propagate at speeds near 1500 m/s, i.e. the speed of sound in water. In this study, we discuss the role of sea-bottom elasticity on the propagation speed of acoustic–gravity waves, and how this alters location calculations of impacting objects. Note that the sound signals that were analysed here are standard sound waves of low frequency nature. They are referred to as acoustic–gravity waves to emphasise that gravitational effects, which can modulate them, are not neglected in the analysis.

**Figure 1:**
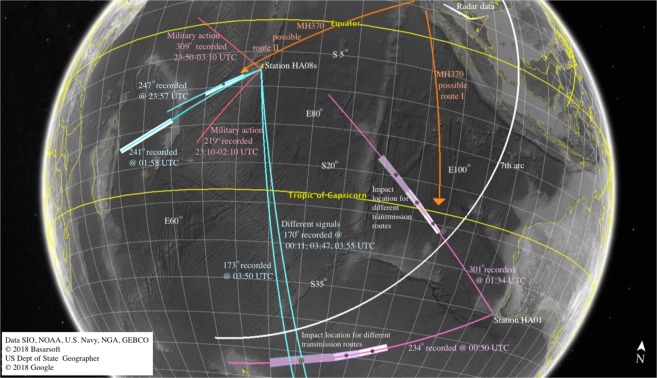
Map of recorded signals on CTBTO hydrophone stations HA01 and HA08s on March 7th and 8th 2014 between 23:00 and 04:00 UTC. Purple: bearing of signals recorded on HA01 that could be associated with MH370, with white-grey polygons that present possible source locations corresponding to transmission routes combining water and sea-bottom following Tables 1 and 2. Cyan: bearing of signals recorded on HA08s that could be associated with MH370, based on Table 3 - bearings 170° and 234° could be related. White: satellite data of the last ‘handshake’ with MH370, known as the 7th arc. Red: bearings of military action that were recorded intermittently on HA08s between 23:00–04:00 UTC. Orange: two possible MH370 routes; only route I is in agreement with the 7th arc. Attribution: Data SIO, NOAA, U.S. Navy, NGA, GEBCO; ©2018 Basarsoft; US Dept of State Geographer; ©2018 Google.

It is now well established that acoustic–gravity waves can travel in the ocean^2–5^ for long distances^6^, yet they can also penetrate through the elastic layers such as sea-bottom^7^ or ice-sheets^8,9^. The transmission mechanism between layers is still not well developed, though it is believed that it could occur once a critical depth (or frequency) is reached^7^. It is also reasonable that transmissions and reflections occur between media (liquid and solid) when a sharp change in the sea-bottom bathymetry is observed, such as a trench, a hill, or a shelf-break^6^. The propagation through multiple layers results in different arrival times, or in the context of this work, would modify the calculated location of impacting objects. To this end, we revisit data that was recorded at the Comprehensive Test Ban Treaty Organisation’s (CTBTO) hydrophone stations HA01 and HA08s on March 7th and 8th 2014 between 23:00 and 04:00 UTC, a time window in which MH370 is believed to have crashed in the Southern Indian Ocean. The findings here not only modify possible impact locations from previously analysed signals on HA01, but also reveal new evidence of missing data on HA08s, which if found related to MH370 would suggest a completely different route and impact location north-east Madagascar, see Fig. 1.

## Results

Upon impact, the generated acoustic–gravity waves radiate in the water layer at about *c*_*l*_ = 1500 m/s. When they penetrate in the solid layer they travel at *c*_*s*_ = 3350 m/s. Acoustic–gravity waves can transmit back and forth from one layer to another, depending on the critical depth/frequency and sharp changes in sea-bottom bathymetry, and that creates a complex matrix of possible routs for the recorded signals as summarised below. In the following we revisit some of the previously analysed signals recorded on station HA01, and introduce new signals recorded on station HA08s. Spectrograms of all signals indicate a significant amount of noise in the 0–4 Hz band. The signals in this band appear to have a broadband frequency content which is typical for low frequency AGWs that can travel long distances before dissipating. We therefore filtered low frequencies (below 5 Hz) with a high pass Butterworth IIR filter. Since the signal randomness measure will change when the signal’s nature changes, transient signals over a noisy background can be identified by calculating a windowed entropy value. Peaks in the entropy trace are present where transient signals are detected. These peaks were considered for the subsequent bearing calculation. After separating the signals, the bearing is calculated using time of arrival based triangulation, see ref.^1^ for detailed bearing calculations.

### HA01

We address two possible impact events that were identified by ref.^1^ thats could be associated with MH370:

1. E1: 301.4 ± 0.4°, 1900 ± 200 km from HA01, centred at −23.662°, 96.676°, recorded at 01:34:40 UTC (event source between 01:11 and 01:16)
2. E2: 234.6 ± 0.4°, 1940 ± 200 km from HA01, centred at −43.487°, 94.469°, recorded at 00:50:00 UTC (event source between 00:25 and 00:31)

**Figure 2:**
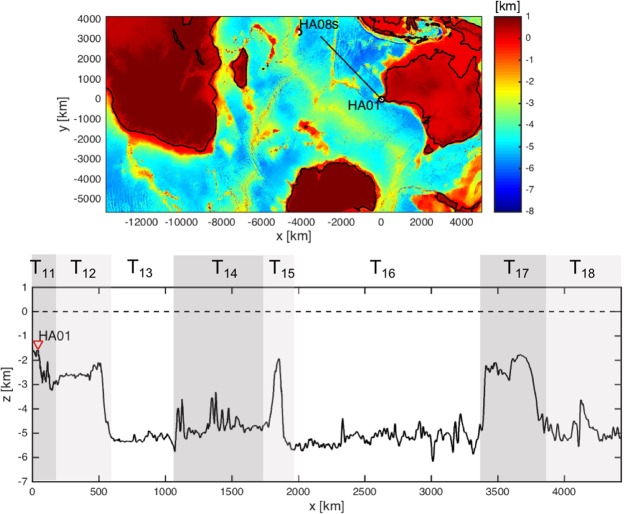
Top: bearing 301° relative to HA01 (solid line). Bottom: transection of sea-bottom, and zones of possible transmissions from and to water or solid layers.

**Figure 3:**
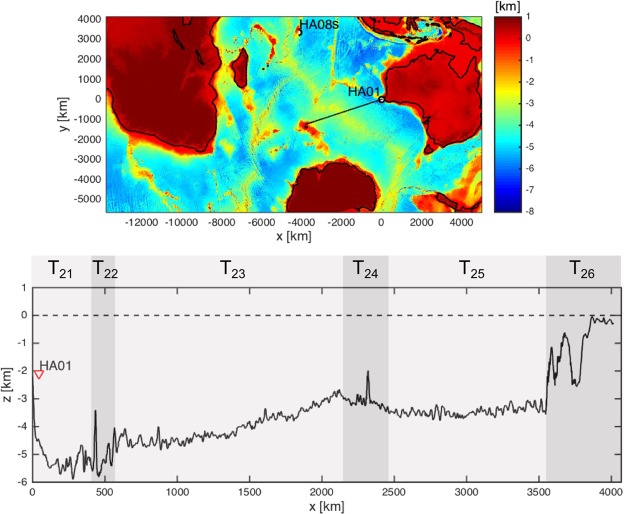
Top: bearing 234° relative to HA01 (solid line). Bottom: transection of sea-bottom, and zones of possible transmissions from and to water or solid layers.

Transects along the bearings of E1 and E2 are given in Figs 2 and 3. Acoustic–gravity waves can transmit between layers at the highlighted regions T_1j_ (j = 1, …, 8) in the case of bearing 301°, and T_2k_ (k = 1, …, 6) in the case of bearing 234°. One possibility, in both E1 and E2, is that the recorded acoustic–gravity waves travelled only in the water layer, which results in the locations originally identified by ref.^1^. Another possibility is that acoustic–gravity waves couple with the elastic layer shortly after the impact and all along the way until the signals are received at the station. This scenario dictates farest location distance that is *c*_*s*_/*c*_*l*_ (almost as twice) the distance originally calculated by ref.^1^. The second scenario is more likely when the water depth at impact is critical. Between these two marginal possibilities there are a number possible transmission between layers that result in different locations, as summarised in Tables 1 and 2.

**Table 1:** Possible transmission routs between water and solid layers for signal E1 on bearing 301°.

Transmission route	Distance (km)	Location
no transmision	1900 ± 200	24°50S 98°06′E
${{\rm{T}}}_{{\rm{16}}}\to {{\rm{T}}}_{{\rm{15}}}$	2226 ± 234	23°01S 95°38′E
${{\rm{T}}}_{{\rm{15}}}\to {{\rm{T}}}_{{\rm{14}}}\to {{\rm{T}}}_{{\rm{12}}}$	2234 ± 235	22°56S 95°35′E
T_15_	2876 ± 303	19°10S 90°55′E
${{\rm{T}}}_{{\rm{16}}}\to {{\rm{T}}}_{{\rm{12}}}$	3664 ± 386	14°18S 85°28′E
T_17_	3918 ± 412	12°43S 83°46′E
T_18_	4294 ± 452	10°21S 81°18′E

*T*_1*j*_ at odd or even sequence order correspond to transmissions from water to solid or solid to water, respectively. The distance is measured from station HA01.

**Table 2:** Possible transmission routs between water and solid layers for signal E2 on bearing 234°.

Transmission route	Distance (km)	Location
no transmision	1940 ± 204	43°29S 94°30′E
${{\rm{T}}}_{{\rm{23}}}\to {{\rm{T}}}_{{\rm{22}}}$	2276 ± 271	44°36S 90°35′E
${{\rm{T}}}_{{\rm{25}}}\to {{\rm{T}}}_{{\rm{24}}}\to {{\rm{T}}}_{{\rm{23}}}\to {{\rm{T}}}_{{\rm{21}}}$	3180 ± 335	46°53S 79°25′E
T_24_	3222 ± 339	46°58S 78°50′E
T_26_	4384 ± 461	48°01S 63°31′E

*T*_1*j*_ at odd or even sequence order correspond to transmissions from water to solid or solid to water, respectively. The distance is measured from station HA01.

### HA08s

**Figure 4:**
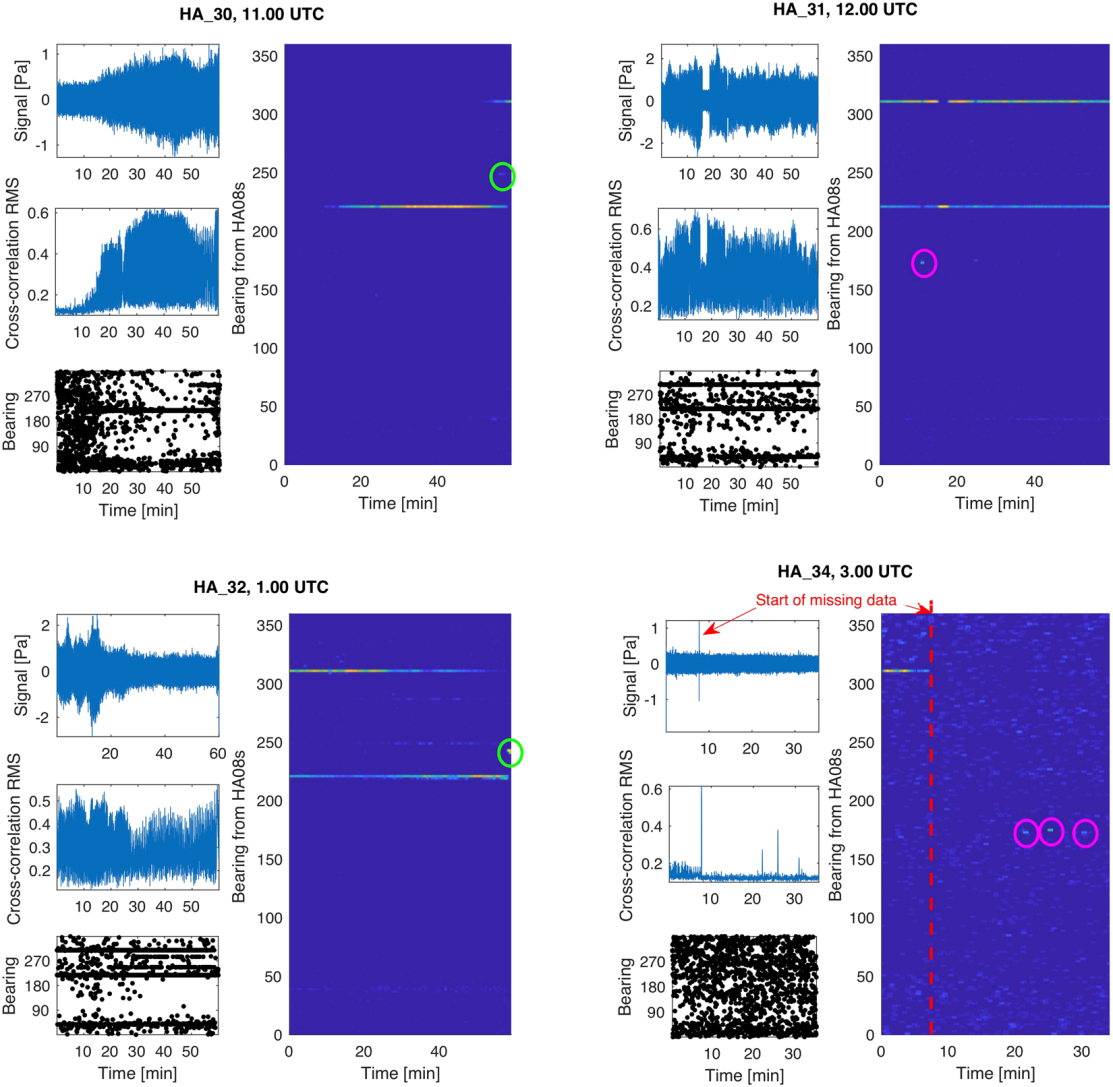
Signal, cross-correlation, and bearings of recordings at HA08s reveals a few signals of interest (summarised in Table 3).

**Figure 5:**
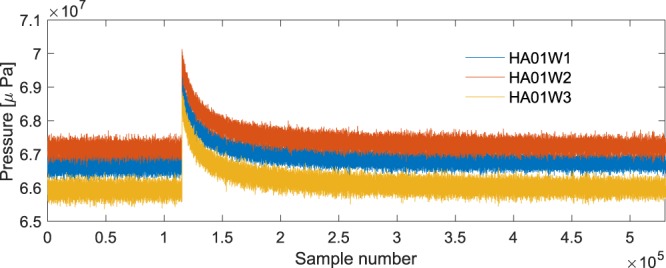
Raw data recorded by all three hydrophones of HA08s. The sharp signal indicates restarting the system after 25 minutes of missing data on all three hydrophones starting from 3:07 UTC.

Analyses of signals recorded at station HA08s (−65.5445°, 32.4730°) were more challenging, partially due to disturbances in the recordings that are believed to be caused by military action in the region. A summary of identified signals of interest (see Fig. 4) is given in Table 3. Note that bearings if signals HA_30 and HA_32 fall within the military action bearings, so it is also possible that the signals are associated with the military action. Among the rest of the signals, it is remarkable that three have a bearing of 170.9°, and one 173°. The first occurred at 12:11 UTC whereas the other three followed about three hours later, all after 3.30. Last but not least, a fifth signal appears at 3:07 (see Fig. 5). This signal probably indicates restarting the system after it was shutdown for 25 minutes, i.e. there is a missing data in these specific CTBTO recordings.

**Table 3:** Signals recorded at HA08s. The recording times are in UTC, and the bearings are relative to HA08s. Signals from military action are intermittent at two locations: 219.2° and 309.7°.

Signal	Time [UTC]	Bearing	Distance [km]	Location
HA_30	11:57	247.4°	585 ± 276	9°34′S 67°36′E
HA_31	12:11	170.9°	2,300 ± 250	28°08′S 76°20′E
HA_32	01:58	241.3°	2,860 ± 900	19°05′S 48°32′E
HA_34a	03:47	170.9°	—	—
HA_34b	03:50	173.0°	—	—
HA_34c	03:55	170.9°	—	—

## Discussion

Acoustic–gravity waves can travel at speeds near the speed of sound in water, yet they can double their speed when coupling with the elastic layer. As they propagate they carry information on their source and thus can be used, among others, for locating impacting objects at the sea surface by applying a proper inverse model^1^. However, since the location directly relies on the propagation speed, and the later depends on the medium, it is important to know the route travelled by acoustic–gravity waves. For example, signals E1 and E2 travel through different routs, as given in Tables 1 and 2, which can results in different calculated locations. In the case of multiple transmissions one expects teh signals to be composed of a number of smaller signals, which is not the case here unless if other signals are buried in the ambient noise. Since only one complete signal has been identified for each of E1 and E2, it is more likely that the signals either did not transmit at all into the elastic layer, or transmitted only once at the initial stage and coupled with the elastic layer all a long the way, i.e. the first and last possibilities of each table.

The locations of signals found on HA08s are with high uncertainty or unknown and require further analysis. Though, if related to MH370 that might suggest a location in the northern part of the Indian Ocean. Due to the sensitivity of the recorded data, it is unlikely that the three hydrophones on HA08s had a simultaneous technical failure and the reason behind the shut down is to-date unknown. The missing data might be related to the military action in the area (during or after the impact), but another argument is that a violent nearby activity (including impact, explosion) could have resulted in a shutdown of the system. Both the signal HA_30 of bearing 247° recorded at 11:57 on March 7th, and the missing data if related to MH370 could (independently) suggest that the impact location is closer to Diego Garcia’s station, as opposed to Cape Leeuwin’s station. With the absence of the recordings, there is currently no scientific evidence that an impact occurred during this time window. However, it might be possible to extract more information after processing hidden signals in the ambient noise. To study this possibility and to further assess the effects of elasticity and transmissions due to sea-bottom topography we intend to carry out a set of field experiments, while in parallel develop a depth-integrated, see^10^, sea-bottom elastic model for the radiation of acoustic–gravity waves from impacting objects.

## Methods

### Dispersion relation

The solution for the propagation of acoustic–gravity waves in compressible water under the effects of gravity, and an elastic half space was treated by ref.^7^, who derived the dispersion relation,

1 $$\tanh (rh)=\frac{\frac{{\omega }^{2}}{r}\{q{\rho }_{l}(\frac{{k}^{2}-{s}^{2}}{{k}^{2}+{s}^{2}})+\frac{1}{g}[\frac{4{k}^{2}qs\mu }{{k}^{2}+{s}^{2}}-(\lambda +2\mu ){q}^{2}+\lambda {k}^{2}]\}}{\frac{{\omega }^{4}q{\rho }_{l}}{g{r}^{2}}(\frac{{k}^{2}-{s}^{2}}{{k}^{2}+{s}^{2}})+[\frac{4{k}^{2}qs\mu }{{k}^{2}+{s}^{2}}-(\lambda +2\mu ){q}^{2}+\lambda {k}^{2}]}$$

where *r* is the eigenvalue, *h* is the water depth, *g* is the acceleration due to gravity, *k* is the wavenumber, *ω* is the frequency, *λ* and *μ* are Lame’s elasticity constants, ${\rho }_{l}$ is the water density, and *q* and *s* are separation constants in the solid sea-bottom,

2 $${r}^{2}={k}^{2}-{\omega }^{2}/{c}_{l}^{2};\,{q}^{2}={k}^{2}-{\omega }^{2}/{c}_{p}^{2};\,{s}^{2}={k}^{2}-{\omega }^{2}/{c}_{s}^{2},$$

where ${c}_{p}=\sqrt{(\lambda +2\mu )/{\rho }_{s}}$ and ${c}_{s}=\sqrt{\mu /{\rho }_{s}}$ are pressure-wave and shear-wave velocities in the sea-bottom, respectively; and ${\rho }_{s}$ is the earth density. For our numerical examples we rely on average parameter-values taken from the entries for the crust and ocean in Table 1 of PREM^11^: ${\rho }_{l}$ = 1020 kg/m^3^, ${\rho }_{s}$ = 2750 kg/m^3^, *c*_*l*_ = 1470 m/s, *c*_*s*_ = 3550 m/s. Employing the dispersion relation allows transmission from the water layer into the elastic sea-bottom and vice-versa. Once coupled with the sea-bottom, the propagation speed increases from *c*_*l*_ to *c*_*s*_. Note that for this main reason, and since we analyse low frequency acoustic–gravity waves that span the entire depth, the effects of stratification and variations of water density on the propagation speed where ignored.

### Impact time and location

The measured frequency of the signal is dependant on the impact time and location, *t*_0_ and *x*_0_ as given by^5,12^:

3 $${\hat{{\rm{\Omega }}}}_{{\hat{t}}_{j}}=\frac{\pi c}{2h\sqrt{1-{[{x}_{0}/c({\hat{t}}_{j}-{t}_{0})]}^{2}}},\,j=\mathrm{1,}\,\mathrm{2,}\,\mathrm{...}$$

By choosing pairs of instants ${\hat{t}}_{m}$ and ${\hat{t}}_{n}$ from the recordings and the corresponding frequencies at these instants ${\hat{{\rm{\Omega }}}}_{{\hat{t}}_{n}}$ and ${\hat{{\rm{\Omega }}}}_{{\hat{t}}_{m}}$, we can solve for *x*_0_ and *t*_0_ explicitly^5^:

4 $${x}_{0}=\frac{({\hat{t}}_{m}-{\hat{t}}_{n})c}{{\{1-{[\pi c\mathrm{/(2}h{\hat{{\rm{\Omega }}}}_{{\hat{t}}_{m}})]}^{2}\}}^{-\mathrm{1/2}}-{\{1-{[\pi c\mathrm{/(2}h{\hat{{\rm{\Omega }}}}_{{\hat{t}}_{n}})]}^{2}\}}^{-\mathrm{1/2}}},$$

and

5 $${t}_{0}={\hat{t}}_{j}-\frac{{x}_{0}}{c}{\{1-{[\frac{\pi c}{2h{\hat{{\rm{\Omega }}}}_{{\hat{t}}_{j}}}]}^{2}\}}^{-\mathrm{1/2}},\,j=n\,{\rm{or}}\,m.$$

**Figure 6:**
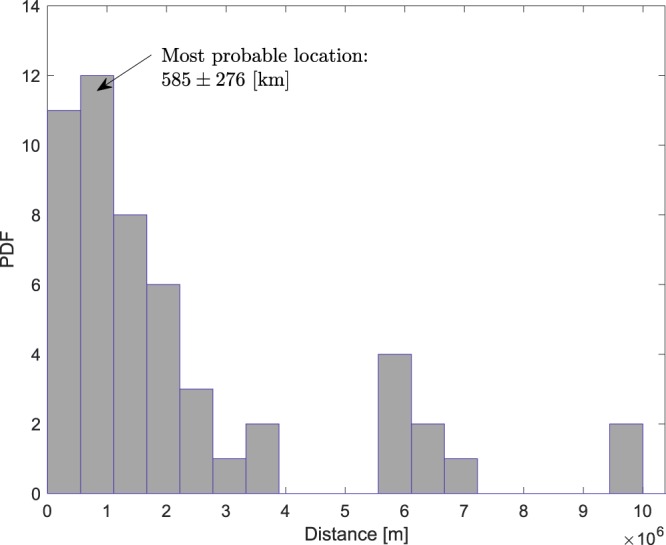
PDF of the location of signal HA_30, recorded at 11:57 UTC, bearing 247.4° from HA08s. The most probable location is at 585 ± 276 km from station HA08s.

For each signal we fix ${\hat{t}}_{n}$ at some instant at the beginning of the signal, and calculate *t*_0_ and *x*_0_ with ${\hat{t}}_{m}$ running from $m=n\,\mathrm{...}\,M$, where *M* denotes the location at the end of the signal. Thus, we obtain a probabilistic distribution of solutions that allow uncertainty about the most probable solution (see Fig. 6). Detailed signals processing and bearing calculations can be found in the methods section of ref.^1^.

## Data Availability

Data can be accessed by direct inquiries to ukadri@mit.edu.
